# De novo* MCM6* variants in neurodevelopmental disorders: a recognizable phenotype related to zinc binding residues

**DOI:** 10.1007/s00439-023-02569-7

**Published:** 2023-05-17

**Authors:** Daphne J. Smits, Rachel Schot, Cristiana A. Popescu, Kerith-Rae Dias, Lesley Ades, Lauren C. Briere, David A. Sweetser, Itaru Kushima, Branko Aleksic, Suliman Khan, Vasiliki Karageorgou, Natalia Ordonez, Frank J. G. T. Sleutels, Daniëlle C. M. van der Kaay, Christine Van Mol, Hilde Van Esch, Aida M. Bertoli-Avella, Tony Roscioli, Grazia M. S. Mancini

**Affiliations:** 1 Department of Clinical Genetics, Erasmus University Medical Center, 3015 GD Rotterdam, The Netherlands; 2 Neuroscience Research Australia (NeuRA), University of New South Wales, Sydney, Australia; 3 Department of Clinical Genetics, The Children’s Hospital at Westmead, Westmead, NSW Australia; 4 Specialty of Genomic Medicine, Faculty of Medicine and Health, University of Sydney, Sydney, NSW Australia; 5 Center for Genomic Medicine, Massachusetts General Hospital, Boston, MA USA; 6 Medical Genomics Center, Nagoya University Graduate School of Medicine, Nagoya, Japan; 7 Department of Psychiatry, Graduate School of Medicine, Nagoya University, Nagoya, Japan; 8 CENTOGENE GmbH, 18055 Rostock, Germany; 9 Department of Pediatrics, Subdivision of Endocrinology, Erasmus University Medical Center, Rotterdam, The Netherlands; 10 Department of Pediatrics, GZ Antwerp, Antwerp, Belgium; 11 Center for Human Genetics, University Hospitals Leuven, 3000 Leuven, Belgium; 12 New South Wales Health Pathology Randwick Genomics, Prince of Wales Hospital, Sydney, Australia; 13 Discovery Unit, Department of Clinical Genetics, Erasmus University Medical Center, 3015 GD Rotterdam, The Netherlands

## Abstract

The minichromosome maintenance (MCM) complex acts as a DNA helicase during DNA replication, and thereby regulates cell cycle progression and proliferation. In addition, MCM-complex components localize to centrosomes and play an independent role in ciliogenesis. Pathogenic variants in genes coding for MCM components and other DNA replication factors have been linked to growth and developmental disorders as Meier–Gorlin syndrome and Seckel syndrome. Trio exome/genome sequencing identified the same de novo* MCM6* missense variant p.(Cys158Tyr) in two unrelated individuals that presented with overlapping phenotypes consisting of intra-uterine growth retardation, short stature, congenital microcephaly, endocrine features, developmental delay and urogenital anomalies. The identified variant affects a zinc binding cysteine in the MCM6 zinc finger signature. This domain, and specifically cysteine residues, are essential for MCM-complex dimerization and the induction of helicase activity, suggesting a deleterious effect of this variant on DNA replication. Fibroblasts derived from the two affected individuals showed defects both in ciliogenesis and cell proliferation. We additionally traced three unrelated individuals with de novo* MCM6* variants in the oligonucleotide binding (OB)-fold domain, presenting with variable (neuro)developmental features including autism spectrum disorder, developmental delay, and epilepsy. Taken together, our findings implicate de novo* MCM6* variants in neurodevelopmental disorders. The clinical features and functional defects related to the zinc binding residue resemble those observed in syndromes related to other MCM components and DNA replication factors, while de novo OB-fold domain missense variants may be associated with more variable neurodevelopmental phenotypes. These data encourage consideration of *MCM6* variants in the diagnostic arsenal of NDD.

**Supplementary Information:**

The online version contains supplementary material available at 10.1007/s00439-023-02569-7.

## Introduction

The minichromosome maintenance (MCM) complex controls early phases of DNA replication and is, therefore, essential for cell proliferation. The MCM complex consists of six subunits (MCM2–7) that form a double hexamer at DNA replication origins. The MCM complex assembles into a pre-replicative complex (Pre-RC) with the origin recognition complex (ORC), CDC6 and CDT1 (Lei 2005; Meagher et al. 2019). During early stages of DNA replication, the MCM complex unwinds double stranded DNA at DNA replication origins, recruits DNA polymerases and initiates DNA synthesis. Because embryonic development requires rapid cell proliferation, it is particularly sensitive to genetic variants that affect DNA replication (Kalogeropoulou et al. 2019; Nordman and Orr-Weaver 2012). Defects in DNA replication affect the number of cells generated during development and are, therefore, related to various human diseases, such as microcephaly and primordial dwarfism (Tingler et al. 2022). In addition, some subunits of the MCM complex localize at the centrosome and are apparently involved in primary cilia homeostasis (Stiff et al. 2013; Casar Tena et al. 2019).

Variants in genes coding for components of the MCM complex, Pre-RC complex and replisome have been linked to various human diseases. Biallelic loss of function (LoF) variants in *MCM3* (Mandelian inheritance in Man (MIM) *602,693), *MCM5* (MIM*602,696), *MCM7* (MIM*600,592), *ORC1* (MIM *601,902), *ORC4* (MIM *603,056), *ORC6* (MIM *607,213), *CDT1* (MIM *605,525)*, CDC45* (MIM*603,465)*,* and *CDC6* (MIM *602,627) have been identified in individuals with Meier–Gorlin syndrome (MGS). MGS is a primordial dwarfism disorder, characterized by proportionate short stature, microtia and patellar hypoplasia, as well as additional variable congenital anomalies (Bellelli and Boulton 2021; Cottineau et al. 2017; Gao et al. 2015; Gineau et al. 2012; Hughes et al. 2012; Knapp et al. 2021; Mace et al. 2020; Vetro et al. 2017; de Munnik et al. 2015). In addition, dominant gain of function variants in *GMNN* (MIM *602,842) are described as cause of MGS (Burrage et al. 2015). Heterozygous variants in *MCM2* (MIM *116,945) are associated with autosomal dominant deafness (Gao et al. 2015). The only member of the MCM2–7 complex for which no pathogenic exonic variants have been described is *MCM6* (MIM *601,806). Intronic variants in *MCM6* have been reported and are associated with lactase persistency via enhanced activation of the nearby lactase gene promotor (Almazar et al. 2019; Jarvela et al. 2009).

While the role of MCM6 in developmental disease is unknown, it does have a well-described role in tumorigenesis (Mughal et al. 2019). A number of studies have shown increased expression of *MCM6* in malignant tumors, such as meningiomas, non-small cell lung carcinoma, chondrosarcoma, endometrial adenocarcinoma and colorectal cancer (Shim et al. 2021; Jia et al. 2020; Issac et al. 2019; Cai et al. 2018; Gauchotte et al. 2012; Hotton et al. 2018). High *MCM6* expression is associated with tumor invasion and the development of metastasis via enhanced tumor cell proliferation rates (Gu et al. 2021; Li et al. 2020). Other studies have shown that downregulation of *MCM6* with siRNA impairs cell cycle progression and cell proliferation, confirming a role for MCM6 in the regulation of cell proliferation rates (Liu et al. 2018). Here, we describe the involvement of de novo* MCM6* variants in neurodevelopmental phenotypes. To support the pathogenicity of one of the identified variants affecting a zinc finger binding residue, we tested the effects of the variant on cell proliferation, ciliogenesis and cell survival.

## Materials and methods

### Consent

The study was approved by the local IRBs (Erasmus MC Rotterdam, protocol METC-2012387, National Institutes of Health, protocol 15HG0130). Written informed consent for participation in the study and scientific publication of imaging data and clinical photographs was obtained. All experiments were performed on fibroblasts sampled for diagnostic procedures. Affected individuals were traced and matched via GeneMatcher and Matchmaker Exchange (Sobreira et al. 2015).

### Data availability

Exome/genome sequencing data are deposited internally in each medical institute, to guarantee privacy of the described individuals. Sequence data for individual 4 are available on dbGaP, per study protocol and consent: phs001232.v1.p1; Seq_DNA_WholeGenome (blood), phs001232.v3.p1; Seq_RNA_Transcriptome (blood).

### Exome sequencing and genome sequencing

Exome sequencing was performed on DNA isolated from blood from probands and family members, as previously described (Vandervore et al. 2019a). Details of sequencing and analysis pipelines are described for each family in the supplemental information.

### Individual 1

Exome sequencing was performed on the Agilent Sure Select platform (Clinical research Exome Capture), run on HiSeq (101 bp paired-end, Illumina), using the diagnostic certified pipeline of the department of Clinical Genetics, ErasmusMC, Rotterdam. The average coverage was ~ 50x. Data were demultiplexed by the Illumina Software CASAVA. Reads are mapped with the program BWA (http://bio-bwa.sourceforge.net/). Variants were detected with the Genome Analysis Toolkit (http://www.broadinstitute.org/gatk/). The Variant Calling File was filtered in Alissa Interpret. Amplification reactions were conducted according to standard methods and purified with ExoSAP-IT (USB). Direct sequencing was performed with Big Dye Terminator chemistry (Applied Biosystems). DNA fragment analysis was performed with capillary electrophoresis on an ABI3130 Genetic Analyzer (Applied Biosystems) with the software package Seqscape (Applied Biosystems). Identified variants were classified based on the guidelines provided by the American College of Medical Genetics and Genomics (ACMG) and Association for Molecular Pathology (AMP) (Richards et al. 2015).

### Individual 2

Exome sequencing was performed at CENTOGENE (accredited diagnostic laboratory) as described previously (Trujillano et al. 2017a). In brief, genomic DNA was enzymatically fragmented, and target regions are enriched using DNA capture probes. These regions include approximately 41 Mb of the human coding exome (targeting > 98% of the coding RefSeq from the human genome build GRCh37/hg19). The generated library is sequenced on an Illumina platform to obtain at least 20 × coverage depth for > 98% of the targeted bases. An in-house bioinformatics pipeline, variant calling, annotation, and comprehensive variant filtering is applied. All variants with minor allele frequency (MAF) of less than 1% in gnomAD database, and disease-causing variants reported in HGMD^®^, in ClinVar or in CentoMD^®^ were evaluated (Trujillano et al. 2017b).

### Individual 3

Trio exome sequencing for individual 3 was completed at NSW Health Pathology Randwick Genomics, Sydney, Australia. DNA was extracted from peripheral blood samples and libraries were prepped for standard ES using Agilent SureSelect QXT CREv2 kit (Agilent Technologies, Santa Clara, CA, USA) with sequencing on an Illumina NovaSeq 6000 (Illumina, San Diego, CA, USA), mapped to hg38/GRCh38 and analyzed using the Illumina DRAGEN Bio-IT Platform. Bioinformatic analyses of SNVs and indels were conducted using the in-house GAIA pipeline (Sundercombe et al. 2021) based on the Gemini v18 platform with annotation from the Variant Effect Predictor (VEP) and dbNSFP under assumptions of Mendelian inheritance. Genomic variants were filtered based on impact, population frequencies of < 2% in the ExAC/gnomAD, 1000 Genomics and laboratory internal databases for homozygous/compound heterozygous models and < 0.1% for de novo heterozygous models. A threshold CADD Phred score of 10 was used as a filter with contribution from other in silico scores, such as SIFT, PolyPhen2 and PROVEAN. The evidence for phenotype-causality was evaluated for each variant resulting from the filtering strategies above and variants were classified utilizing the American College of Medical Genetics and Genomics and the Association for Molecular Pathology (ACMG–AMP) guidelines (Richards et al. 2015) with modifications (Amendola et al. 2016; Nykamp et al. 2017; Strande et al. 2017), incorporating aspects of the scoring system reported by Karbassi et al. (2016).

### Individual 4

Quad WGS (proband, unaffected parents, unaffected sibling) was performed by Hudson Alpha Clinical Services Laboratory using the Illumina HiSeq X sequencing platform. The clinical analysis by Hudson Alpha did not yield any variants that were felt to be likely to explain the individual’s neurodevelopmental phenotype. Research-based reanalysis of the data was performed by Brigham Genomic Medicine, leading to the identification of the *MCM6* variant of interest (Haghighi et al. 2018). Sanger confirmation of this variant for the family of four was performed by Hudson Alpha.

### Individual 5

Trio exome sequencing was performed and described in Takata et al. (2018).

### Protein modelling

The PyMOL Molecular Graphics systems was used to visualize the 3D protein The PyMOL model of the MCM hexamer complex. The protein data bank ID (PDB-ID) of the protein model used is 7W1Y.

### Cell culture

Fibroblasts derived from punch biopsies of the skin, sampled for diagnostic purposes, were grown in full medium [Dulbecco’s Modified Eagle Medium (DMEM) with 10% fetal bovine serum (FBS) and 1% penicillin/streptomycin (v/v)] at 37 °C and 5% CO_2_. Cells were enzymatically detached using trypsin/EDTA (0.25%).

### Cilia number and length

Fibroblasts were plated (5 × 10^4^ cells) on 24 mm cover slips and cultured in full medium overnight. The following day, media was replaced with serum-free medium for 48 h. Cells were fixed with 4% paraformaldehyde (PFA) for 20 min on ice. Samples were blocked on ice for 1 h in blocking buffer containing 50 mM Tris HCl [pH 7.4], 0.9% NaCl, 0.25% gelatin and 0.5% Triton X-100. Primary antibodies were dissolved in blocking buffer. Sections were incubated overnight at 4 °C. The next day slides were washed three times with PBS and incubated with the secondary antibodies for 1 h at RT. Coverslips were mounted with DAPI containing Prolong Gold (ThermoFisher Scientific).

Primary antibodies used: mouse monoclonal anti-human acetylated tubulin (Sigma Aldrich^®^, T7451, ICC 1:8000), Rabbit polyclonal anti-human gamma tubulin (Sigma Aldrich^®^, T3320, ICC 1:1000). Secondary antibodies used: Cy™3 AffiniPure Donkey Anti-Mouse IgG (H + L) (1:200, Fisher Scientific, 715–165-150), Cy™5 AffiniPure Donkey Anti-rabbit IgG (H + L) (1:200, Fisher Scientific, NC0470376).

### Cell cycle analysis

Fibroblasts were grown to 70–80% confluence in T75 flasks. After detachment, cell suspensions were diluted till a final concentration of 3–4 × 10^5^. After centrifugation, cells were fixed in 200ul 4% PFA for 30 min on ice. Fixation was followed by a washing step with 10% FCS–PBS and permeabilization in 10% FCS–PBS supplemented with 0.1% Triton for 1 h on ice. Pellets were washed with 10% FCS–PBS and afterwards dissolved in 2 ug/ml Hoechst 33,342. Hoechst was incubated for 1 h at 37 °C and 1 h at room temperature, after which the samples were analyzed using the BD LSRFortessa Cell Analyser. FlowJo^®^v.7.6.5 was used for analysis.

### EdU proliferation assay

Fibroblasts (1.5 × 10^5^) were grown on 24 mm coverslips. The cells were incubated with 10 µM EdU solution in complete media for 24 h. After incubation, the cells were fixed in 4% PFA. Cells were permeabilized in 0.1% PBS-Triton for 20 min on ice. During permeabilization, the Click-iT reaction cocktail mix was prepared following manufacturer’s instructions (The Click-iT EdU assay 647; Thermo Fisher Scientific, USA) and incubated for 30 min at RT. The coverslips were mounted using 20 µl ProLong Gold antifade reagent with DAPI (Thermo Fisher Scientific, USA).

### FLICA apoptosis assay

Fibroblasts were grown till 60–70% confluence. Cell lines were treated with 0.5 mM H2O2 dissolved in DMEM with 10% FCS for 2 h at 37 °C. Afterwards, cells were washed with PBS and enzymatically detached with trypsin–EDTA. The FAM-FLICA^®^ Caspase-3/7 Assay Kit was used to quantify apoptotic cells, following manufacturer’s instructions with minor adaptions. Cell suspensions were diluted to 4 × 10^5^/mL and incubated with the 30 × FLICA stock (1:30) in an Eppendorf tube for 1 h at 37 °C. After incubation with FLICA, cells were washed once with 10% FCS–PBS, and incubated for 1 h in DMEM+/+. Afterwards, cells were centrifuged and co-stained with propidium iodide (PI) (0.5%v/v) for 5 min at room temperature. The cell pellet was diluted to a final volume of 500ul for analysis with flow cytometry. Cells were analyzed with the BD LSRFortessa Cell Analyser, counting at least 20.000 events per sample within 1 h after staining. The 530/30 laser was used to excite FAM–FLICA dye and the 610/20 laser was used to excite PI. FlowJo®v.7.6.5 was used for analysis.

### Confocal microscopy

Confocal fluorescent images of cells were acquired with the Broadband Leica TCS SP5, using Leica LAS AF software (Leica Microsystems). Lasers with 405 nm, 561 nm or 633 nm excitation wavelength were used to visualize DAPI, cy3 and cy5 secondary antibodies. All images were processed and blindly analyzed with Fiji ImageJ software.

### Statistics

All statistical tests were performed with Prism GraphPad 9 Software. All data sets were tested for outliers and normality. All error bars represent the standard error of the mean (SEM). Details about the statistical tests are available in the figure legends.

## Results

De novo variants in *MCM6* were identified in five affected children from five unrelated families. The clinical features from all affected individuals are summarized in Table 1. Extensive case reports and detailed clinical and genomic information are provided in the supplemental material and Table S1.

**Table 1:** Clinical features observed in individuals with a de novo* MCM6* variant

	Individual 1	Individual 2	Individual 3	Individual 4	Individual 5
de novo* MCM6* variant(NM_005915.5)	c.473G > A, p.(Cys158Tyr)	c.473G > A, p.(Cys158Tyr)	c.605A > G, p.(Asp202Gly)	c.715G > A, p.(Gly239Ser)	c.445C > T, p.(Pro149Ser)
Protein domain	Zinc finger residue	Zinc finger residue	OB-fold	OB-fold	OB-fold
CADD score	29.5	29.5	29.1	33	28
Birth weight	– 3.9 SD	– 3.3 SD	– 1.5 SD	+ 1.09 SD	− 1.2 SD
Length at birth	– 5.7 SD	– 6.2 SD	– 3 SD	+ 1.4 SD	0 SD
OFC at birth (SD)	– 5.5 SD	– 5.9 SD	– 2 SD	+ 1 SD	− 0.8 SD
IUGR	Yes	Yes	Yes	No	No
Developmental delay	Yes	Yes	Yes	Developmental regression	Mild
Endocrine disorders	Premature adrenarche, advanced bone age	Pseudo-hypoaldosteronism	No	Hirsuitism, elevated DHEAS and testosterone	–
Urogenital/kidney anomalies	Undescended testes	Microgenitalia	No	No	No
Epilepsy	No	No	No	Yes	No
ASD	No	Yes	Yes	Yes	Yes

*ASD* autism spectrum disorder, *DHEAS* dehydroepiandrosterone sulfate, *IUGR* intra-uterine growth restriction, *OB-fold* oligonucleotide binding-fold, *OFC* occipitofrontal circumference

### Variants in zinc binding residues

**Fig. 1:**
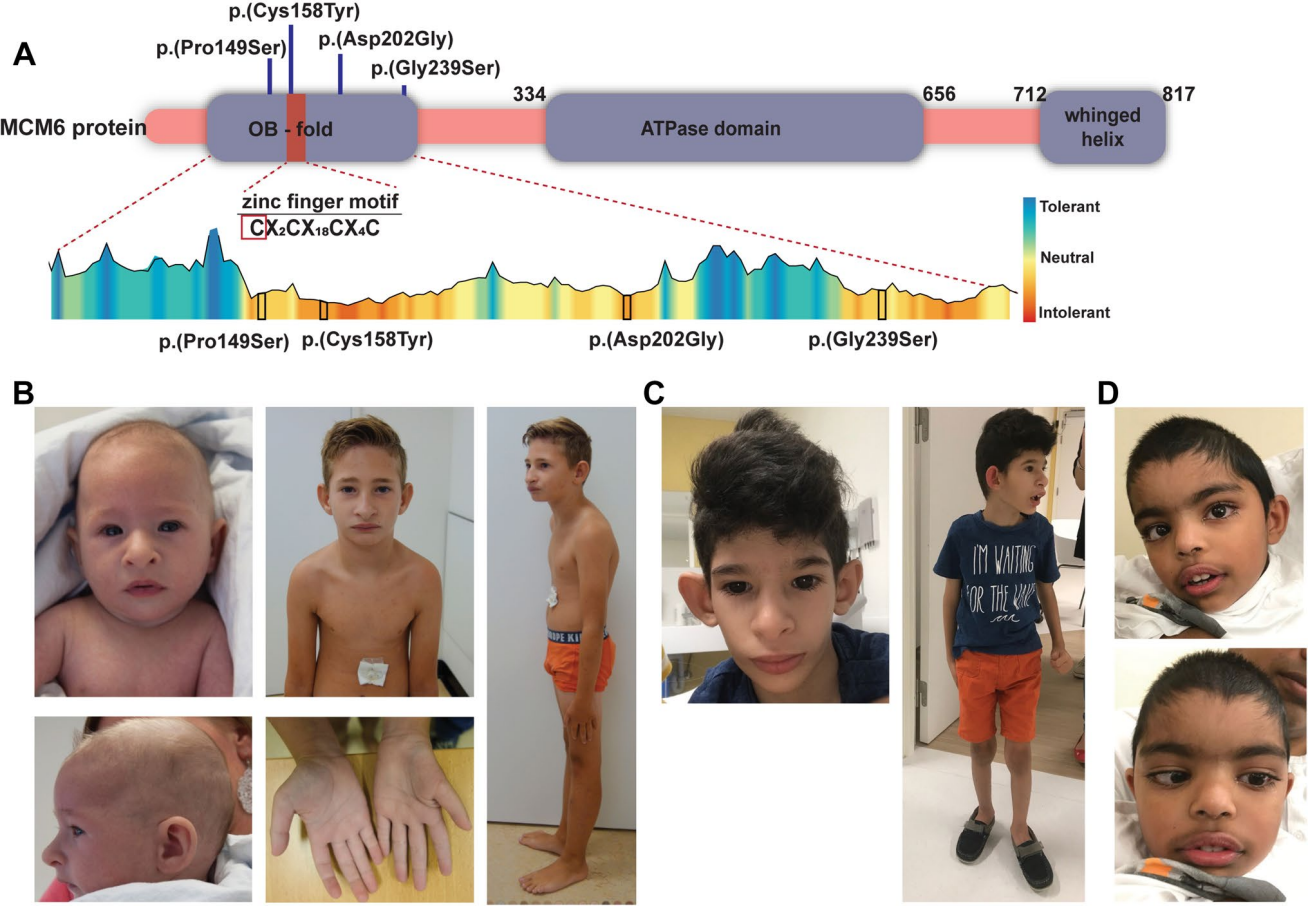
Clinical phenotype of individuals with *MCM6* variants. **A** Schematic overview of the identified *MCM6* variants depicted in the MCM6 protein structure, based on NM_005915.5. Tolerance data for the MCM6 OB-fold and zinc finger domain extracted from the MetaDome database (Wiel et al. 2019). Missense variants identified in the affected individuals are depicted in black boxes. **B** Facial features and posture of individual 1 at 4 months of age (left upper and lower panels) and 8 years of age (middle and right panels). **C** Facial features (left) and posture (right) of individual 2 at 6 years of age. **D** Facial features individual 3 at 4 years of age (upper and lower right panel)

In the affected individuals 1 and 2, we identified the same de novo missense variant in *MCM6*. This heterozygous missense variant of uncertain significance (VUS Class 3; NM_005915.5 (*MCM6*): c.473G > A, p.(Cys158Tyr)), which we will subsequently indicate as p.(Cys158Tyr), affects a cysteine residue in the zinc finger domain (Fig. 1A). This variant affects a highly conserved residue, with deleterious effects on protein functioning (according to MutationTaster, PolyPhen-2, SIFT) and a CADD score of 29.5. This variant has never been reported in gnomAD v2/v3.

*Individual 1* was born from unrelated healthy Dutch parents. Intra-uterine growth restriction (IUGR) was noticed during pregnancy. He was born small for gestational age (SGA) after 37 + 5 weeks of gestation with low birth weight (− 4.27 SDS), short length (− 5.05 SDS) and severe microcephaly (− 4.24 SDS). After birth, he was diagnosed with neonatal sepsis. From birth, he had persistent feeding difficulties for which he received permanent gastric tube feeding. He has mild developmental delay, mostly affecting speech, and attends special primary education. His facial features include a receding forehead, brachycephaly, short palpebral fissures, large prominent nose, flared nostrils, large ears, receding chin, malocclusion and a prominent upper lip. Kyphosis, a small thorax, a proximally implanted thumb, hypoplastic thenar eminence, a sandal gap and undescended testes were present at physical examination (Fig. 1B). He demonstrated spontaneous catch-up growth after birth until a height of approximately − 2 SDS at the age of 4 years (Fig. S1). He developed pubic hair at the age of 7.5 years with a bone age that is advanced by 4 years. Laboratory investigations including a high-dose adrenocorticotropic hormone (ACTH) stimulation test demonstrated some elevated steroid precursors without consistent evidence for an adrenal enzymatic defect. No abnormalities in genes associated with congenital adrenal hyperplasia were identified. SNP array and screenings for metabolic disorders were normal.

The second individual with the p.(Cys158Tyr) variant (Individual 2) presented with very similar clinical features compared to individual 1. This boy was born in Venezuela from unrelated healthy parents. From the 20th week of gestation, severe growth retardation was noted for which a caesarian section was performed at the 35th gestational week. He was born with low birth weight (− 3.3 SDS), short length (− 6.2 SDS) and severe microcephaly (− 5.9 SDS). After birth he presented with neonatal sepsis, persistent foramen ovale, micropenis and cryptorchidism. During the first years of life adrenal insufficiency was suspected and he was treated with hydrocortisone. His speech development was severely delayed while his motor abilities were relatively good. At last examination, at 6 years of age, he was still non-verbal but understood simple tasks and used sign language. Weight gain was slow, his height was a -4 SDS and the OFC at -7 SDS. Treatment with hydrocortisone was ceased when the endocrine diagnosis was revised to pseudo-hypoaldosteronism. A diagnosis of autism spectrum disorder was made and he developed febrile seizures. His facial features include small face with protruding ears, broad nose with bulbous tip and flared nostrils, full prominent lips, large frontal incisors, epicanthal folds and long eyelashes (Fig. 1C). Micro-array analysis for genomic copy number variants was negative.

### Variants in OB-fold

In three additional unrelated individuals (3, 4 and 5) different de novo missense variants were identified in the oligo nucleotide binding (OB)-fold domain. (Fig. 1A). Details about the individual variants are available in Table 1, Table S1 and the Supplemental information. These variants were classified as putative pathogenic because of high CADD scores (range 28–33) and because they have never been reported in gnomAD v2/v3. They were predicted to have damaging effects by SIFT, deleterious effects by MutationTaster and probably damaging effects by PolyPhen-2.

Clinically these three individuals show a variable neurodevelopmental phenotype that only partially overlaps with the features observed in the individuals with variants in the zinc binding residue. Individual 3 presented with intra-uterine growth restriction, borderline primary microcephaly and severe/moderate ID. He was born prematurely to healthy, but short (length at 1st %), parents from Bangladesh. He was born by caesarean section for fetal bradycardia to an insulin-dependent diabetic mother. He had a length and head circumference well below average for gestational age (− 3SDS length, − 2SDS OFC) (Supplemental material). After birth he presented with hypertonia and lower limb spasticity. Concerns about his development were raised by second year of life when he did not start walking. He could crawl and just pull to stand and had no words. He was diagnosed with autism spectrum disorder (ASD) and had rigid behavior and echolalia. He has small hands and feet, and mild thoracic kyphosis. His facial features include craniofacial dysmorphism with low anterior hairline, unusual hair pattern, large looking eyes, long palpebral fissures, hypertelorism, right convergent strabismus, mild synophrys, long eyelashes, broad nasal bridge, large teeth, short philtrum, prominent lips, and over-folded ear helices (Fig. 1D). Micro-array was negative, but trio exome sequencing detected a de novo* MCM6* variant (NM_005915.5 (*MCM6*): c.605A > G, p.(Asp202Gly)).

Despite normal initial development, the affected individual 4 presented with developmental regression in early childhood, following a series of difficult to treat urinary tract infections. She has been diagnosed with autism and severe intellectual disability and is non-verbal. She has infrequent epileptic seizures, which are typically triggered by prolonged periods of poor sleep. Signs of autonomic instability with severe small fiber neuropathy were observed. She showed recurrent fungal infections that may be at least partially attributable to myeloperoxidase deficiency. Moreover, recurrent moderate hyperammonemia was observed, that is thought to represent mild OTC deficiency, although a causative *OTC* (OMIM *300,461) variant has not been identified. The affected individual was born at term following an unremarkable pregnancy, and birth weight was + 1.15 SDS. Length and head circumference measurement from birth were not available. At 10 years of age weight, length, and head circumference were at + 0.39 SDS, + 0.36 SDS, and + 1.3 SDS, respectively. The affected girl subsequently developed significant obesity, and weight, length, and head circumference measurements at 25 years of age were + 4.38 SDS, -1.17 SDS, and + 2.11 SDS, respectively. The affected individual is non-dysmorphic. Quad genome sequencing detected a de novo* MCM6* variant (NM_005915.5 (*MCM6*): c.715G > A, p.(Gly239Ser)). Several additional variants, including known pathogenic variants in *MPO* (MIM *606,989), were identified in this individual and mentioned in the supplemental results.

The last affected individual (individual 5) presented with a de novo variant in *MCM6* (NM_005915.5 (*MCM6*): c.445C > T, p.(Pro149Ser)) and was previously reported in a large study cohort on autism spectrum disorders (Huang et al. 2022). This boy had normal birth parameters and postnatal growth. During development, he showed mild delay in language development. At follow-up, aged 5 years, he had autism spectrum disorder, absent eye contact, echolalia, poor response to name calling, fascination with lights and hyperarousal to sensory input. He had visual anomalies which were characterized by poor eyesight, no eye pursuit, nystagmus, strabismus, and a suspected retinal tumor. Since this individual is no longer followed in the corresponding clinic, additional details about his medical records are not available.

### Protein modelling

**Fig. 2:**
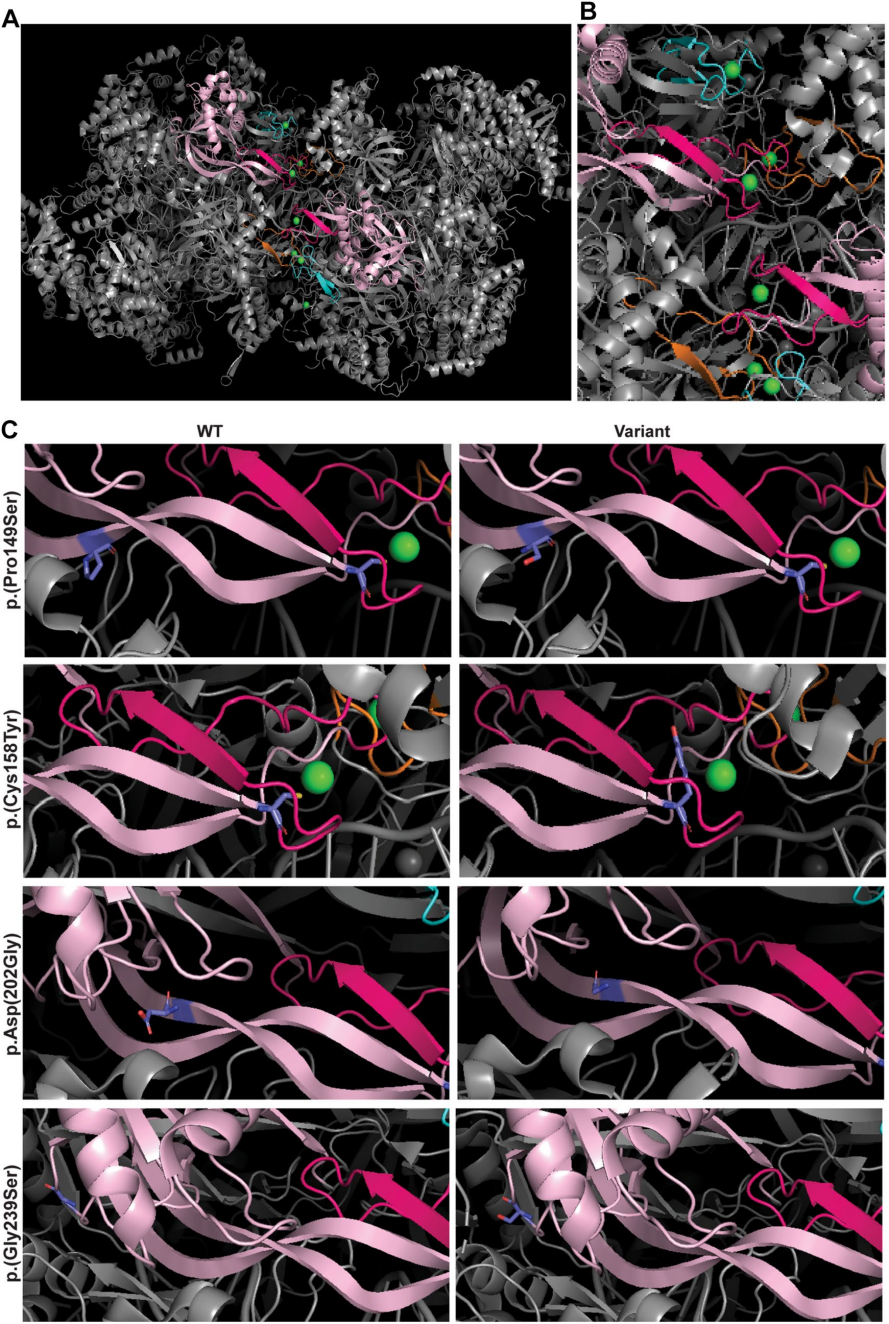
3D modeling of the MCM complex dimer and the identified variants. **A** 3D-model of the MCM hexamer complex visualized in PyMOL Molecular Graphics (PDB: 7W1Y). The zinc finger domains are depicted in orange (MCM2), blue (MCM4) and pink (MCM6) The MCM6 OB-fold domain is depicted in light pink. The zinc atoms are depicted as green balls. **B** Inset hexamer dimer interface showing the close proximity between the zinc finger domains of MCM2, MCM4 and MCM6. **C** Left panels show the WT amino acid residue (purple), the substitution as observed in the affected individuals is shown in the right panels

The p.(Cys158Tyr) variant, identified in individuals 1 and 2, affects a zinc binding residue in the zinc finger domain of MCM6. Zinc finger domains are involved in the establishment of protein–protein interactions and protein–DNA/RNA interactions. In the MCM complex, the zinc finger residues are directed towards the dimer interface and are involved in hexamer dimerization (Fig. 2A, B). Zinc finger domains typically contain 4 cysteine or histidine residues, which can bind Zn (Meagher et al. 2019^)+^ ions. The variant identified in both affected individuals from family 1 and 2 (NM_005915.5 *MCM6*: c.473G > C, p.(Cys158Tyr)) changes the first cysteine residue of this domain into a tyrosine residue. Since tyrosine cannot bind Zn^2+^ ions, this variant most likely impairs zinc binding and disturbs the protein/DNA binding capacities of this domain (Fig. 2B, C/Fig. S2).

The variants identified in individuals 3–5 are located in the OB-fold domain (Fig. 1A). The OB-fold binds single stranded DNA and mainly consists of β-sheets that are flanking the zinc finger domain. All missense variants identified in the OB-fold alter the amino acid hydrophobicity and polarity and could thereby affect local protein structure and nucleic acid interaction (Bischoff and Schluter 2012; Kyte and Doolittle 1982). The p.(Pro149Ser) and p.(Asp202Gly) variants are located in one of the β-sheets flanking the zinc finger domain and might affect the rigidity of this β-sheet as both serine and glycine are flexible amino acids (Fig. 2C/Fig. S2). The p.(Gly239Ser) variant affects a glycine located in a loop connecting two β-sheets, in the core of the protein. This variant induces loss of a flexible glycine in this loop (Fig. 2C/Fig. S2). Fibroblasts derived from individuals with OB-fold domain variants were not available for additional functional studies.

### The p.(Cys158Tyr) zinc finger variant impairs cell proliferation and ciliogenesis

**Fig. 3:**
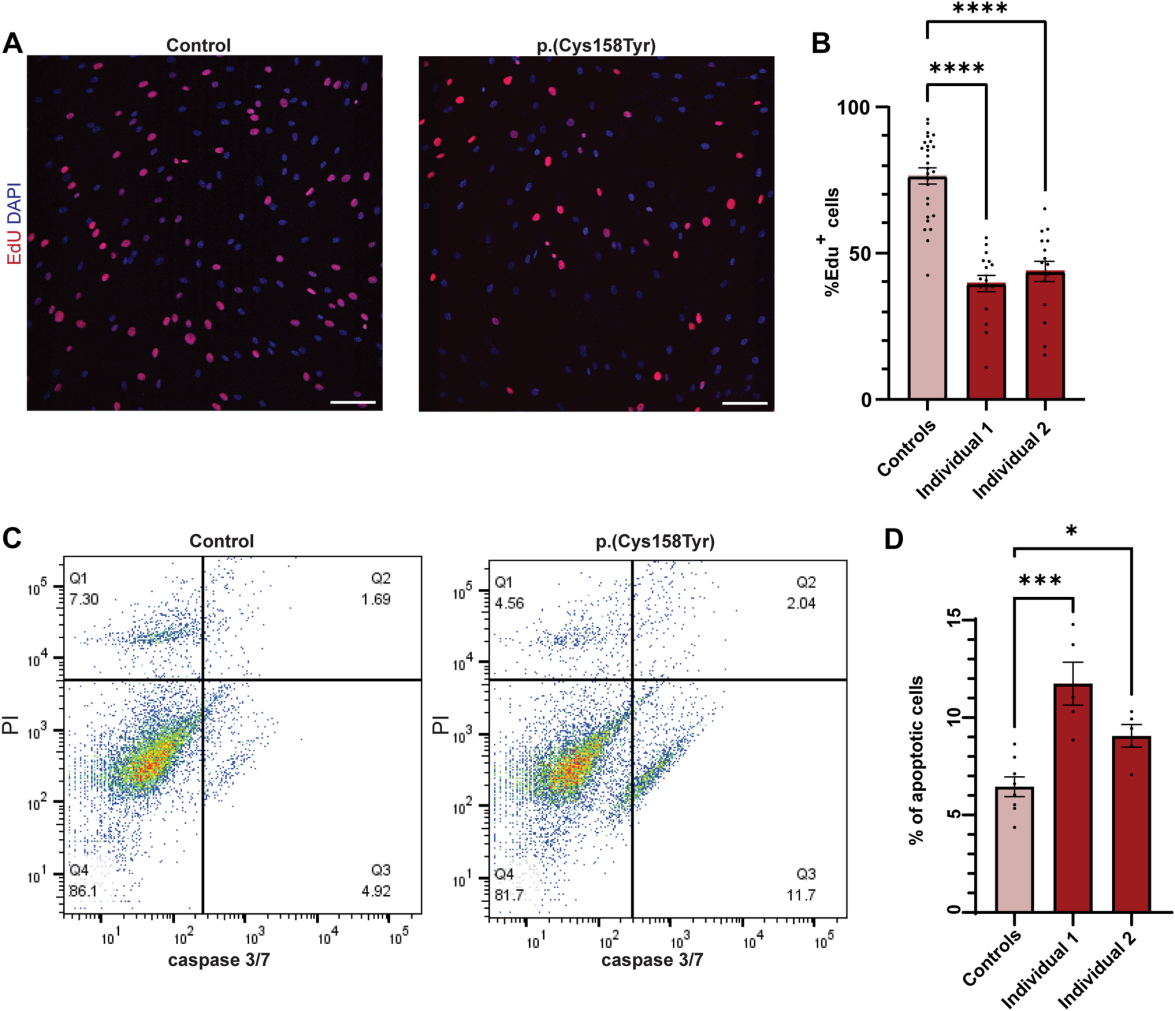
Effect of the zinc finger variants on cell proliferation and apoptosis. **A** Click-IT EdU proliferation assay was performed on fibroblasts from affected individuals 1 and 2, and 2 independent control fibroblast lines. Cells that did incorporate EdU during incubation are shown in red, all nuclei were counterstained with DAPI (blue). Scale bars indicate 20 µm. **B** Quantification of the percentage of proliferating fibroblasts. The number of EdU + (red) cells was calculated as a percentage of all DAPI + (blue) nuclei (One-way ANOVA, ****p < 0.0001, mean ± SEM, *n* =  > 8–10 fields per slide, *n* = 3 experiments). **C** Number of apoptotic and necrotic cells was determined with flow cytometry after staining with FAM–FLICA and propidium iodide (PI). Cells were sorted on apoptotic signal intensity (FAM–FLICA/caspase 3/7) and necrotic signal intensity (PI), which resulted in the formation of four clusters. Cluster Q1 depicts necrotic cells, Q2 shows late apoptotic cells, Q3 shows early apoptotic cells and Q4 shows healthy cells. **D** Number of apoptotic cells (Q2 + Q3) was calculated as a percentage of all cells analyzed (*n* = 3 experiments, *n* =  > 10.000 cells per analysis, One-way ANOVA: *p = 0.034, ***p = 0.0002, mean ± SEM)

Regulation of DNA replication by the MCM complex is essential during embryonic development. Defects in DNA replication impair cell proliferation rates and induce apoptosis. Since these processes significantly alter the number of progenitor cells during early developmental stages, they are known causes of congenital microcephaly and primordial dwarfism (Kalogeropoulou et al. 2019; Bellelli and Boulton 2021; Gu et al. 2021). In the human brain, MCM genes have the highest expression levels in the first 10 weeks of gestation, supporting their involvement in neuronal progenitor proliferation (Fig. S3A + B). To explore the effect of the MCM6 p.(Cys158Tyr) variant to cell proliferation, we assessed the proliferating capacities of fibroblasts from individuals 1 and 2 by quantifying EdU incorporation (Fig. 3A). Both fibroblast lines containing the MCM6 p.(Cys158Tyr) variant showed a significant decrease in EdU positive cells compared to age and sex matched control fibroblast lines, confirming decreased proliferation rate in vitro **(**Fig. 3B) (control: 76.3%, p.(Cys158Tyr): individual 1: 39.6%, individual 2: 43.6%, One-way ANOVA, ****p < 0.0001). Increased susceptibility of p.(Cys158Tyr) fibroblasts to apoptosis was evaluated by detecting CASPASE 3/7 levels after stress induction by hydrogen peroxide. Both MCM6 p.(Cys158Tyr) cell lines showed a significant increase in the percentage of cells that expresses caspases 3/7 (Q2 + Q3 Control:6.45%, individual 1: 11.7%, individual 2: 9.1) compared to controls (Fig. 3C, D). This increase was mainly due to an increase of cells in early apoptosis (Q3).

**Fig. 4:**
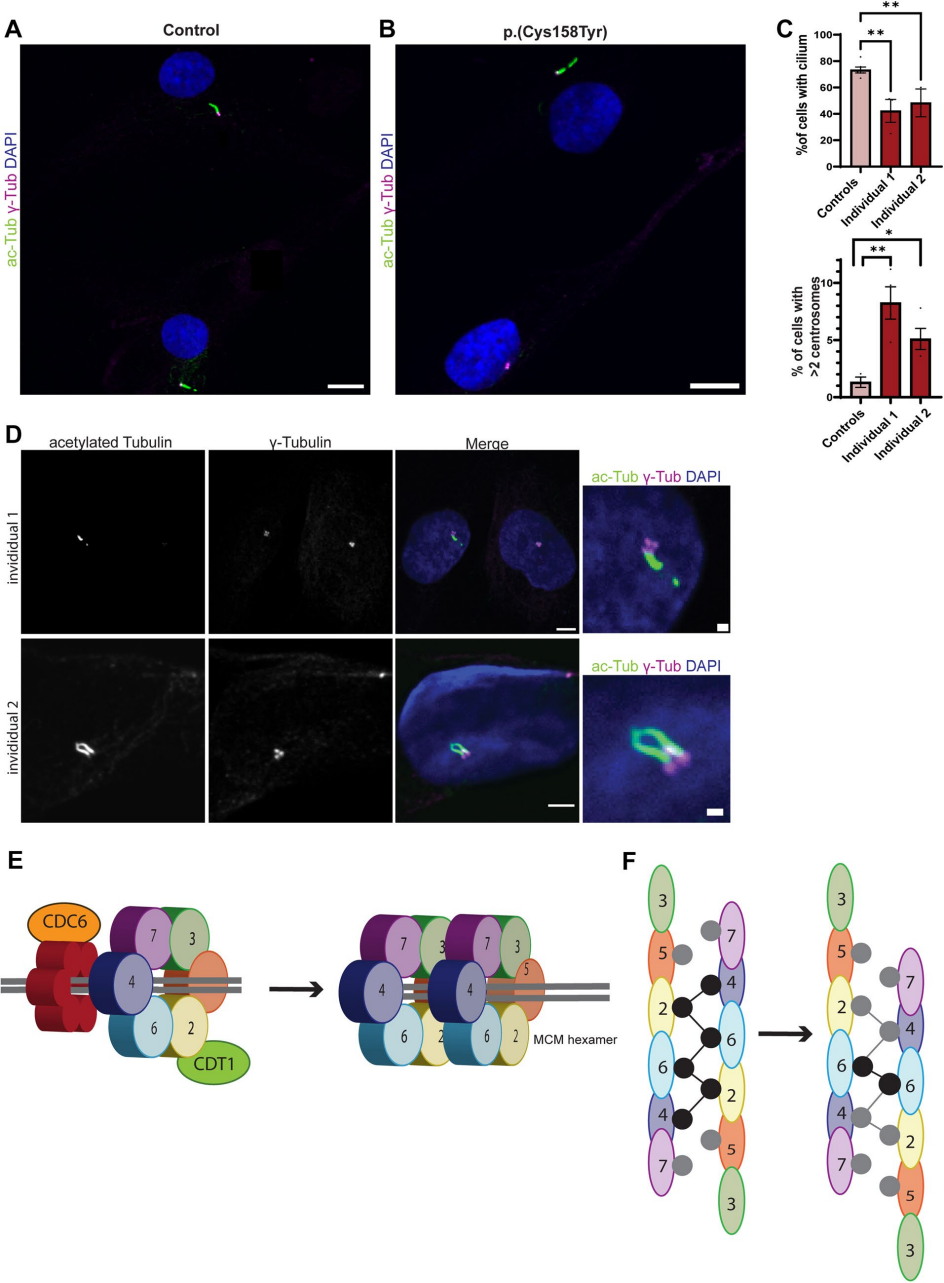
Zinc finger variant impairs cilia formation and increases the number of centrosomes. **A** Ciliogenesis was evaluated in fibroblasts from affected individual 1 and 2 and two control fibroblast lines. Cells were starved for 48 h after which the number of cells containing a primary cilium was evaluated by staining with acetylated tubulin (green) and centrosomal marker γ-Tubulin (magenta). Nuclei were co-stained with DAPI. **B** Quantification of A. At least 100 cells were analyzed per data point (*n* = 4 experiments, *n* = 2 controls, One-way ANOVA: **(individual 1) *p* = 0.0026, **(individual 2) *p* = 0.0096, mean ± SEM). Scale bars indicate 20 μm. **C** Percentage of cells with > 2 centrosomes during interphase was quantified. At least 100 nuclei were counted per data point (*n* = 2 experiments, *n* = 2 controls, One-way ANOVA: *p = 0.047, **p = 0.0017, mean ± SEM). **D** Fibroblasts were stained with the cilia marker acetylated Tubulin (green), centrosome marker γ-Tubulin (magenta) after 48 h of starvation. Nuclei were co-stained with DAPI. Examples of fibroblasts derived from individuals 1 and 2 (p.(Cys158Tyr)) with multiple centrosomes and 2 cilia are shown. Scale bars in this figure indicate 5 μm, or 1 μm in insets. **E** Schematic illustration showing recruitment of the MCM hexamer complexes and CDT1 to DNA replication origins on double stranded DNA. The MCM complex assembles into a pre-replicative complex with the origin recognition complex (ORC), CDC6 and CDT1(left). After the recruitment of two MCM hexamers, these hexamers form a homodimer (right panel). **F** Schematic illustration of the double MCM hexamer formation by zinc finger domains. The initial double helix (left panel) undergoes a one-subunit shift at the dimer interaction interface to promote conversion to the dCMGE complex. Gray and black circles represent zinc fingers mediating the interaction between the two hexamer complexes. Black circles indicate zinc fingers involved in tight inter-ring interactions, adapted from Lewis et al. (2022)

In addition to their well-established role in DNA replication, MCM and ORC components are also involved in cilia formation and centrosome duplication (Stiff et al. 2013). Cell lines from MGS patients, harboring variants in ORC and MCM genes, show defects in centrosome number and delays in cilia formation (Casar Tena et al. 2019). MCM6 has been shown to be part of the centrosomal proteome and has been detected at the centrosomes (Andersen et al. 2003; Stuermer et al. 2007). Therefore, we hypothesized that the MCM6 p.(Cys158Tyr) variant might disturb centrosome-directed assembly of primary cilia during interphase. To study ciliogenesis, fibroblasts derived from affected individuals were synchronized by serum starvation, as described previously (Vandervore et al. 2019b). Both p.(Cys158Tyr) cell lines showed a remarkable decrease in number of cells that were able to build a primary cilium (Fig. 4A). Where about 75% of age and sex matched control fibroblasts did develop a primary cilia, only 42% and 48% of the cell lines with the p.(Cys158Tyr) variant showed cilia, independently of cilium length (Fig. 4B). Moreover, a subfraction of p.(Cys158Tyr) cells showed supernumerous (> 2) centrosomes (individual 1: 8.25%, individual 2: 5.1%), which was rarely observed in control cell lines (1.3%) (Fig. 4C, D). Sporadically, we observed multiple cilia in the p.(Cys158Tyr) cell lines, a feature which was never seen in control lines (Fig. 4C). These results support that the p.(Cys158Tyr) zinc finger variant leads to loss of global MCM complex function, including its functions related to DNA replication as well as its functions related to cilia homeostasis.

## Discussion

Here we present five individuals from five unrelated families with de novo missense variants in the zinc finger and OB-fold domain of *MCM6*, presenting with variable neurodevelopmental features depending on the affected functional protein domain. The performed functional studies are consistent with a crucial role for MCM6 zinc binding residues in the regulation of cell proliferation and cilia formation.

### Variants in Zinc binding residues

The two affected individuals with the zinc binding residue variant p.(Cys158Tyr), have similar clinical features including primary microcephaly, developmental delay, typical facial characteristics, endocrine features, feeding difficulties and urogenital anomalies. These clinical features show overlap with those observed in other DNA replication disorders, such as Meier–Gorlin syndrome (MGS) and Seckel syndrome (SS) (Tingler et al. 2022; Bellelli and Boulton 2021; de Munnik et al. 2012, 2015). Both SS and MGS are associated with variants in genes encoding pre-replication complex components (Bellelli and Boulton 2021; de Munnik et al. 2015). Moreover, biallelic variants in the MCM hexamer components *MCM5* and *MCM7* are associated with MGS (Knapp et al. 2021; Vetro et al. 2017). Therefore, we consider that the features observed in individuals with the MCM6 zinc finger p.(Cys158Tyr) variant, fit in the phenotypic spectrum described for MCM complex related disorders.

These two individuals presented with severe growth delays and primary microcephaly, suggesting that the MCM6 zinc finger domain is crucial for cell proliferation. In vitro, the zinc finger variant indeed caused a remarkable decrease in proliferation rate. Moreover, these cells showed enhanced susceptibility to apoptosis. These observations support that this variant induces a loss of MCM6 function, as siRNA mediated knockdown of *MCM6* also reduces cell proliferation rates and increases the number of apoptotic cells (Gu et al. 2021; Li et al. 2020). In addition, deficiency of proteins from the pre-RC complex, such as ORC1/ORC4/ORC6/MCM2/MCM7/CDT1/CDC6, affects ciliogenesis and leads to delayed cilia formation, shorter cilia, increased centrosome numbers or multiple cilia. The ciliogenesis defects observed in the MCM6 p.(Cys158Tyr) cell lines show high overlap with those described anomalies and, therefore, support the pathogenic effect of this heterozygous variant (Stiff et al. 2013; Casar Tena et al. 2019; Maerz et al. 2019).

Recently, the zinc finger domain of MCM6 was reported to fulfill an essential role in MCM complex activation during DNA replication (Lewis et al. 2022). Upon the formation of the Pre-RC complex, two MCM2–7 hexamers must form a homodimer (Fig. 4E). Afterwards, additional proteins (CDC45, GINS, POLε) bind to the dimer to form the dCDC45–MCM–GINS–POLε (dCMGE) complex. The zinc finger domains of MCM2–7 are directed towards the hexamer interface and mediate the hexamer dimerization step (Fig. 4F). After dimerization, a conformational change of the double hexamer is needed to enable binding of GINS (Lewis et al. 2022). During the conformational shift, some zinc fingers interactions are lost, but others remain tethered (Fig. 4F right panel). Remarkably, the MCM6 zinc finger seems to be crucial for this transition as it is one of the few zinc fingers that remains tightly bound upon this shift (Lewis et al. 2022).

While variants in other MCM complex components have been related to MGS-like phenotypes or natural-killer-cell (NK-cell) deficiency, all of those reported have been biallelic (Gineau et al. 2012; Hughes et al. 2012; Knapp et al. 2021; Vetro et al. 2017). Interestingly, none of the previously identified MCM variants was located in a MCM zinc finger binding residue, nor in the protein regions flanking the zinc finger domains. The affected zinc finger is a highly conserved domain and mutations in the MCM zinc finger motif of *Methanobacterium thermoautotrophicum* have dominant negative effects on cell survival and are defective in the assembly of the pre-RC complex (Fletcher et al. 2005).

While the exact effect of the identified p.(Cys158Tyr) MCM6 zinc finger variant on hexamer dimerization, the conformational change and MCM complex activation in human cell lines remains unexplored, we hypothesize that this variant also has a dominant negative effect on hexamer dimerization. The heterozygous variant will be incorporated in about 50% of the MCM hexa-monomers. Upon dimerization, more than 50% of the MCM double hexamer complexes will contain at least 1 mutant hexamer, hence influencing the function of the bound WT hexamer and explaining the pronounced clinical phenotype of this heterozygous variant. This could represent a more general phenomenon: it is possible that deleterious de novo variants in zinc finger domains of other MCM components that are involved in homodimer formation and the following conformational shift (e.g., MCM2, MCM4), could be related to autosomal dominant phenotypes.

In vitro, the zinc finger variant caused a remarkable decrease in proliferation rate, enhanced the susceptibility to apoptosis, and impaired ciliogenesis. These observations support that this variant induces a significant loss of MCM6 functioning, as siRNA mediated knockdown of *MCM6* reduces cell proliferation rates and increases the number of apoptotic cells as well (Gu et al. 2021; Li et al. 2020). While defects in cell proliferation and apoptosis are well-known mechanisms underlying microcephaly, the observed defects in ciliogenesis are quite unusual and not frequently observed (O'Neill et al. 2018). Instead, the in vitro depletion of other MCMs/ORCs has been shown to result in similar defects including impaired/delayed cilia formation, the presence of shorter cilia, increased centrosome numbers or multiple cilia (Stiff et al. 2013; Casar Tena et al. 2019; Maerz et al. 2019).

While MCM6 has previously been described to interact with and localize at centrosomes, its function at these organelles is unclear (Andersen et al. 2003; Stuermer et al. 2007). It is not clear whether the MCM6-related ciliogenesis defect that we observed is secondary to the centrosomal abnormalities or in itself contributes to the phenotype. The clinical spectrum of cilia-related disorders is extremely broad and ever changing (Focsa et al. 2021). In particular, brain development seems to depend on intact centrosome-directed cilia structure and function, during both neuronal proliferation and migration steps (Wilsch-Brauninger and Huttner 2021; Zaidi et al. 2022). Studies in human disorders have associated centrosomal abnormalities with microcephaly (Wilsch-Brauninger and Huttner 2021; Youn and Han 2018), and in some cases with effects on in vitro ciliogenesis (Vandervore et al. 2019b).

### Variants in OB-fold domain

The three additional individuals (individual 3, 4 and 5) with putative pathogenic variants in *MCM6* presented with more variable (neuro)developmental phenotypes including developmental delay/regression, autism spectrum disorders, epilepsy and hypertonia. The variants identified in these individuals were located in the OB-fold domain. OB-folds are involved in oligonucleotide binding and consist of at least five β-strands that form a β-barrel. The β-barrel can be opened or closed when loops like the β-strands adopt their conformation (Murzin 1993; Nguyen et al. 2020; Flynn and Zou 2010). The p.Asp202 and p.Pro149 residues are located in two of the β-strands that are known to bind to ssDNA (Fig. S4). As both observed human variants are predicted to induce changes in hydrophobicity, these variants might influence DNA binding capacities. The p.Gly239 residue is located in one of the loops linking two β-strands. However, the function of this loop and its flanking sheets is less characterized.

Individual 4, harboring the p.(Gly239Ser) variant, has a rather complex phenotype and presents with a number of phenotypic features that are not observed in the other individuals. Most likely, she has several genetic contributors explaining her phenotype. The identified pathogenic *MPO* variants are a known cause of myeloperoxidase deficiency and may contribute to the recurrent fungal infections (Nauseef et al. 1994; Marchetti et al. 2004). However, despite extensive genetic testing, no variants in candidate genes that could potentially explain her autism spectrum disorder were identified, other than the variant in *MCM6*.

Features of ASD were also observed in all other individuals with OB-fold domain variants. While the relation between the MCM complex and ASD has only poorly been described, de novo variants in *MCM6*, *MCM4* and *MCM2* have been reported previously in large ASD cohort studies. Individual 5 from this report was previously described in Takata et al., who implicated *MCM6* as a strong candidate ASD gene after being significantly enriched in ASD cohorts with damaging de novo variants (Takata et al. 2018; Iossifov et al. 2014). De novo missense variants in *MCM4* are related to ASD, and more recently de novo variants in *MCM2* were found in children with sensory processing dysfunction and ASD (Neale et al. 2012; Marco et al. 2018). However, recent cumulative cohort data has not confirmed *MCM6* among major—or moderate ASD risk genes (Satterstrom et al. 2020; Zhou et al. 2022). Given the phenotypic diversity observed in individuals 3,4, and 5, additional reports and functional studies are required to define the functional consequences of these variants at molecular level and their interactions with other MCM components or DNA, and their contribution to the observed phenotypes.

## Conclusion

Our observations support a role for *MCM6* during embryonic (brain) development. We observed a recognizable clinical phenotype, including primary microcephaly, short stature, endocrine features and developmental delay, in individuals with a *MCM6* variant affecting the zinc binding residue. This variant most likely results in a dominant negative effect on MCM6 complex dimerization and activation (Fig. S4), explaining the severe effects of this heterozygous variant. In addition, we identified three individuals with a de novo* MCM6* variant in the OB-fold domain. These individuals shared neurodevelopmental features, including ASD and delayed speech development. Therefore, we suggest to consider de novo* MCM6* variants in individuals with variable neurodevelopmental features and growth abnormalities.

### Web resources

gnomAD https://gnomad.broadinstitute.org/

BrainSpan http://www.brainspan.org/

Human and Mouse Development: http://genebrowser.unige.ch/humous/.

Alphafold: https://alphafold.ebi.ac.uk/

GeneMatcher: https://genematcher.org/statistics/.

## Supplementary Information

Below is the link to the electronic supplementary material.

Supplementary file1 (DOCX 3064 KB)

Supplementary file2 (XLSX 25 KB)

## Data Availability

Exome/genome sequencing data are deposited internally in each medical institute, to guarantee privacy of the described individuals. Sequence data for individual 4 is available on dbGaP, per study protocol and consent: phs001232.v1.p1; Seq_DNA_WholeGenome (blood), phs001232.v3.p1; Seq_RNA_Transcriptome (blood).
