# Supplementary material for: De novo MCM6 variants in neurodevelopmental disorders: a recognizable phenotype related to zinc binding residues
Source: Hum Genet. 2023 May 17;142(7):949–64. doi: 10.1007/s00439-023-02569-7 (PMC10329600; doi:10.1007/s00439-023-02569-7)
Supplement: Supplementary file 1 — Supplementary file1 (DOCX 3064 KB) [file 439_2023_2569_MOESM1_ESM.docx]

Supplementary material - *De novo* *MCM6* variants in neurodevelopmental disorders: a recognizable phenotype related to zinc binding residues

Daphne J. Smits,^1*^ Rachel Schot,^1^ Cristiana A. Popescu,^1^ Kerith-Rae Dias,^2^ Lesley Ades,^3,4^ Lauren C. Briere,^5^ David A. Sweetser,^5^ Itaru Kushima,^6^ Branko Aleksic,^7^ Suliman Khan,^8^ Vasiliki Karageorgou,^8^ Natalia Ordonez,^8^ Frank J.G.T. Sleutels,^1^ Daniëlle C.M van der Kaay,^9^ Christine Van Mol,^10^ Hilde Van Esch,^11^ Aida M. Bertoli-Avella,^8^ Tony Roscioli,^2,12^ Grazia M.S Mancini^1^

Corresponding author: [d.smits@erasmusmc.nl](mailto:d.smits@erasmusmc.nl)

**Supplemental material and methods**

*Whole exome sequencing*

Individual 1: Exome sequencing was performed on the Agilent Sure Select platform (Clinical research Exome Capture), run on HiSeq (101bp paired-end, Illumina), using the diagnostic certified pipeline of the department of Clinical Genetics, ErasmusMC, Rotterdam. The average coverage was ~50x. Data was demultiplexed by the Illumina Software CASAVA. Reads are mapped with the program BWA (http://bio-bwa.sourceforge.net/). Variants were detected with the Genome Analysis Toolkit (http://www.broadinstitute.org/gatk/). The Variant Calling File was filtered in Alissa Interpret. Amplification reactions were conducted according to standard methods and purified with ExoSAP-IT (USB). Direct sequencing was performed with Big Dye Terminator chemistry (Applied Biosystems). DNA fragment analysis was performed with capillary electrophoresis on an ABI3130 Genetic Analyzer (Applied Biosystems) with the software package Seqscape (Applied Biosystems).

Individual 2: Exome sequencing was performed at CENTOGENE (accredited diagnostic laboratory) as described previously^31^. In brief, genomic DNA was enzymatically fragmented, and target regions are enriched using DNA capture probes. These regions include approximately 41 Mb of the human coding exome (targeting > 98% of the coding RefSeq from the human genome build GRCh37/hg19). The generated library is sequenced on an Illumina platform to obtain at least 20x coverage depth for > 98% of the targeted bases. An in-house bioinformatics pipeline, variant calling, annotation, and comprehensive variant filtering is applied. All variants with minor allele frequency (MAF) of less than 1% in gnomAD database, and disease-causing variants reported in HGMD®, in ClinVar or in CentoMD® were evaluated ^32^.

Individual 3: Trio exome sequencing for individual 3 was completed at NSW Health Pathology Randwick Genomics, Sydney, Australia. DNA was extracted from peripheral blood samples and libraries were prepped for standard ES using Agilent SureSelect QXT CREv2 kit (Agilent Technologies, Santa Clara, CA, USA) with sequencing on an Illumina NovaSeq 6000 (Illumina, San Diego, CA, USA), mapped to hg38/GRCh38 and analyzed using the Illumina DRAGEN Bio-IT Platform. Bioinformatic analyses of SNVs and indels were conducted using the in-house GAIA pipeline ^33^ based on the Gemini v18 platform with annotation from the Variant Effect Predictor (VEP) and dbNSFP under assumptions of Mendelian inheritance. Genomic variants were filtered based on impact, population frequencies of <2% in the ExAC/gnomAD, 1000 Genomics and laboratory internal databases for homozygous/compound heterozygous models and <0.1% for *de novo* heterozygous models. A threshold CADD Phred score of 10 was used as a filter with contribution from other *in silico* scores such as SIFT, PolyPhen2 and PROVEAN. The evidence for phenotype-causality was evaluated for each variant resulting from the filtering strategies above and variants were classified utilizing the American College of Medical Genetics and Genomics and the Association for Molecular Pathology (ACMG-AMP) guidelines ^34^ with modifications ^35-37^, incorporating aspects of the scoring system reported by Karbassi *et al*.^38^.

Individual 4: Quad WGS (proband, unaffected parents, unaffected sibling) was performed by Hudson Alpha Clinical Services Laboratory using the Illumina HiSeq X sequencing platform. The clinical analysis by Hudson Alpha did not yield any variants that were felt to be likely to explain the individual’s neurodevelopmental phenotype. Research-based reanalysis of the data was performed by Brigham Genomic Medicine, leading to the identification of the *MCM6* variant of interest ^39^. Sanger confirmation of this variant for the family of four was performed by Hudson Alpha.

Individual 5: Trio exome sequencing was performed and described in Takata *et al* ^40^.

**Supplemental results**

*Individual 1 -* *Clinical report*

The affected individual from this family is the first child born from unrelated healthy parents. During pregnancy intra-uterine growth restriction was noted. He was born with caesarian section at 37+5 weeks of gestation due to nuchal cord. He was born small for gestational age with a birth weight of 1570 grams (-4.27 SDS), birth length of 39 centimeters (-5.05 SDS) and was microcephalic (OFC: 27.8cm, -4.24 SDS). After birth he suffered from enterococcus sepsis which was treated with intravenous antibiotics. He was noted to have dysmorphic features (prominent nose, small chin, protruding ears, low set hear line, deep set eyes, short philtrum, retrognathia) and cryptorchidism. From birth onwards, he had a mild global developmental delay. From birth he developed progressive feeding difficulties for which he required nasogastric feeding and underwent percutaneous endoscopic gastrostomy and Nissen fundoplication together with bilateralorchidopexy at the age of 1.5 years. MRI brain made at two months of age showed no major structural abnormalities besides a thin corpus callosum and mild simplification of the gyral pattern. He demonstrated spontaneous catch-up growth after birth until a height of approximately -2 SDS at the age of 4 years. He developed pubic hair at the age of 7.5 years with a bone age that is advanced by 4 years. There were no signs of early central puberty (no penile enlargement or increase in testicular volume). Laboratory investigations including a high-dose ACTH stimulation test demonstrated elevated dehydroepiandrosterone sulphate, corticosterone, 11-desoxycortisol and androstenedione levels without consistent evidence for an adrenal enzymatic defect. Genes associated with congenital adrenal hyperplasia (*CYP11A1, CYP11B1, CYP17A1, CYP21A2* and *HSD3B2*) were analyzed with detail, but no pathogenic variants were found. This resulted in the diagnosis of premature adrenarche. An LHRH test confirmed the lack of central start of puberty.

*Genomic analysis*

SNP array was normal. Screening for metabolic disorders was normal. Trio exome sequencing identified a de novo nonsynonymous missense heterozygous variant (NM_005915 (*MCM6*): c.473G>A p.(Cys158Tyr)). This variant affects a highly conserved amino acid and is predicted to be deleterious by SIFT, probably damaging by PolyPhen-2 and disease causing by MutationTaster. The CADD score is 29.5, not reported in gnomAD. In addition, ES trio revealed compound heterozygous variants of unknown significance (VUS) in *CFAP61*. These variants are known to cause male infertility, but they are not related to the features described here.

*Individual 2 - Clinical report*

The affected individual was born in Venezuela from unrelated healthy parents. From the 20^th^ week of gestation, severe growth retardation was noted for which a caesarian section was performed at the 35^th^ gestational week. He was born dysmature with severe microcephaly (birth weight 1.040 kg, -3.3SD; length 32 cm, -6.2SD; head circumference 23 cm, -5.9SD). After birth he presented with neonatal sepsis, persistent foramen ovale and microgenitalia (micro-penis, cryptorchidism). During the first years of life adrenal insufficiency was suspected for which hydrocortisone therapy was initiated. His speech development was severely delayed while his motor abilities were relatively good. At last examination, 6 years of age, he was still non–verbal but understands simple tasks and uses sign language. He showed dystrophic growth and insufficient weight gain. Treatment with hydrocortisone was ceased because of a more recent diagnosis of pseudo-hypoaldosteronism. In the meantime a diagnosis of autism spectrum disorder was made and he also developed febrile seizures. Growth measurements at last examination (6y) were: length 100 cm (-4SD), head circumference: 40 cm (-7SD). He goes to a school for children with special needs. His facial features include small face with protruding ears, broad nose with bulbous tip and flared nostrils, full lips, large frontal incisors, epicanthal folds and long eyelashes.

*Genomic analysis*

Micro-array, gene panel analyses for primordial dwarfism and primary microcephaly were all negative.

The exome was performed by Centogene and identified the same de novo missense variant in *MCM6* as found in individual 1 (NM_005915 (*MCM6*): c.473G>A, p.(Cys158Tyr)).

*Individual 3 -* *Clinical report*

This boy was born from healthy Bangladeshi parents at 33 gestational weeks by low segment caesarean section for fetal bradycardia. He was small for gestational age prenatally, and mother had insulin-dependent diabetes during the pregnancy. He was born at BW 1.98kg, L 38cm (1^st^), HC 27cm (1^st^). No resuscitation was needed. He was in special care nursery for temperature regulation and feeding, for about 8 weeks. He had coarse facial features as a neonate and a congenital hernia. Concerns about his development were raised by second year of life when he didn’t start walking. He could crawl and just pull to stand and had no words. In addition, he had feeding difficulties and was unable to tolerate solids. He started walking alone at 3 years 1 month and had 10-120 words by 3 years 3 months. By then, he was not toilet trained and had severely affected language skills.

Physical examination showed increased tone and deep tendon reflexes in lower limbs. He presented with a variable increase in tone of the upper limbs (tone issues improved with time). He had moderate to severe global developmental delay, autism spectrum disorder, relative short stature (but both parents are short with heights on 1^st^ centile). In 2019, his measurements were HC 50.2cm (50th percentile), height 93.5cm (<1^st^ percentile), weight 14.8kg (10th percentile). He developed other problems including echolalia, tooth grinding, rigid behaviour and a disturbed sleep pattern. He requires assistance with all activities of daily living and receives early intervention, speech physiotherapy and occupational therapies.

His facial features include craniofacial dysmorphisms with low anterior hairline, unusual hair pattern with hair extending forwards onto forehead and temporal regions, large looking eyes, long palpebral fissures (>+2SD), measurable hypertelorism (IP on 97^th^ centile), right convergent strabismus, mild synophrys, long eyelashes, broad nasal bridge, large teeth, short philtrum, fullish lips (like his father) and over-folded ear helices. In addition, he has small hands and feet, brachydactyly and broad feet with prominent heels. The mid-finger - and hand lengths both measured <3rd centile; foot length measured between 3^rd^-25th centile. He has normal male external genitalia, a mild thoracic kyphosis and a short neck. Additional investigations showed normal results for creatine kinase (CK), thyroid function tests (TFTs), Eucs, vitamin B12 and D, ferritin, lipid profile, liver function tests, UMS and urinary glycosaminoglycans (GAGs). Cerebral MRI was normal.

*Genomic analysis*

Micro-array was negative.

Exome sequencing identified a de novo missense variant in *MCM6* (NM_005915 (*MCM6*): c.605A>G, p.(Asp202Gly). His variant has CADD Phred score of 29.0. Prediction programs report damaging effects (MutationTaster: deleterious, PolyPhen-2: probably damaging, SIFT: deleterious).

*Individual 4 – clinical report*

The individual 4 is a 39-year-old female. She was born at 40 weeks gestation following an uncomplicated pregnancy and delivery, and her neonatal history was unremarkable. Her Apgar scores were 8 and 9, birth weight was 3926 g (+1.09 SD), and birth length was 53.34 cm (+1.08 SD). While head circumference at birth was not noted, her head circumference at two months of age was 40.32 cm (+1.08 SD).

Her initial development was unremarkable, without any reported delays. She experienced chronic diaper rash in infancy. Starting at 22 months of age she had a series of difficult to treat urinary tract infections without significant fever. Her language development plateaued in this time frame, followed by regression, and she has been non-verbal since approximately three years of age and was subsequently diagnosed with autism. A neuropsychological assessment at age 32 years indicated severe intellectual disability with very limited receptive language and adaptive skills, attention deficits, motor delays, absence of expressive language, and symptoms consistent with a diagnosis of autism spectrum. Throughout her life, the affected individual has continued to experience urinary tract infections and recurrent fungal infections. The fungal infections may be at least partially attributable to myeloperoxidase deficiency (diagnosed at 25mo, biallelic pathogenic *MPO* variants subsequently identified).

From early childhood, the affected individual’s mother noted self-restriction of protein, and the affected individual has a long history of recurrent moderate hyperammonemia. Ornithine transcarbamylase (OTC) levels on liver tissue biopsy were lower than average, in the range expected for a carrier of OTC deficiency, while carbamoylphosphate synthase 1 levels where just slightly below average. While a diagnosis of mild OTC deficiency is suspected, a causative variant has not been identified in *OTC* or any other known urea cycle disorder gene.

The affected individual has experienced recurrent episodes of severe pain since early childhood. In some cases, the pain was attributed to severe headaches, while at other times it was felt to be gastrointestinal or gynecologic in origin. She has had severe constipation and gastrointestinal (GI) dysmotility, resulting in frequent hospitalizations. She underwent multiple rectal biopsies, some but not all of which showed aganglionosis. The affected individual ultimately underwent a partial colectomy at age 16, resulting in improvement of her constipation.

Beginning at approximately 12 years of age, the affected individual experienced significant and unexplained weight gain, and she’s been obese since this time, with BMIs as high as 46 kg/m^2^. At this time the affected individual also began experiencing episodes of cyclic vomiting, which have persisted to the present and have resulted in multiple hospitalizations. The affected individual had her first seizure, a generalized tonic-clonic seizure, at age 17 years. She’s experienced rare seizures since that time, frequently triggered by lack of sleep. The affected individual has autonomic instability, manifested by urinary retention, GI dysmotility, cyclic vomiting, and neuropathic pain. At age 37, a skin punch biopsy at the distal leg revealed severe local sensory and autonomic axonopathy.

*Genomic analysis*

Affected individual 4 has had extensive genetic testing throughout her life. She’s had a normal karyotype, normal FISH for the 15q11-q13 and 17p11.2 regions, multiple normal chromosomal microarrays, negative mitochondrial genome sequencing and deletion testing, and negative fragile X syndrome testing. Dedicated sequencing of the *MECP2*, *SLC25A13*, *OTC*, *CPS1*, *NAGS*, *TTR* (exons 2-3), and *RRM2B* genes was negative. Research-based exome sequencing, the details of which were not available, was reportedly non-diagnostic. Subsequent clinical proband-only exome sequencing revealed two heterozygous pathogenic variants in *MPO* (NM_000250.2 (*MPO*): c.1705C>T, p.(Arg569Trp)) and NM_000250.2 (*MPO*): c.2031-2A>C, p.?) and a single heterozygous pathogenic variant in *BCKDHB* (NM_000056.4 (*BCKDHB*): c.884delT, p.(Leu296fs)). No second variant was identified in *BCKDHB*, and the affected individual has normal allo-isoleucine levels. Quad genome sequencing, performed by Hudson Alpha’s Clinical Services Laboratories in conjunction with the affected individual’s participation in the Undiagnosed Diseases Network Study, identified four variants. The previously identified *MPO* variants were confirmed to be in trans and were felt to be a potential contributor to the affected individual’s history of recurrent fungal infections. A single heterozygous variant of uncertain significance in *PGAP2* (NM_001256237.1 (*PGAP2*):c.320C>T, p.(Thr107Met)) was not felt to be relevant, nor was a single heterozygous pathogenic variant in *ADAR* (NM_015840.4 (*ADAR*): c.577C>G, p.(Pro193Ala)).

Research-based reanalysis of the quad genome sequencing data highlighted three additional findings of potential interest, and the variants were Sanger confirmed in the affected individual and family members in clinical lab. Compound heterozygous missense variants in *MYO16* (NM_015011.3 (*MYO16*):c.2486A>G, p.(Tyr829Cys)) and (NM_015011.3 (*MYO16*):c.3772G>A, p.(Ala1258Thr)), both predicted benign according to the known databases, were identified in the affected individual, her unaffected brother carried only one of the two variants. *MYO16* is a gene with no known disease association. The affected individual was also found to have two *de novo* variants, a rare synonymous variant in *ZNF516* (NM_014643.3 (*ZNF516*): c.2808C>T, p.(Ile926Ile)), and a missense variant in *MCM6* (NM_005915.5 (*MCM6*): c.715G>A, p.(Gly239Ser))

**Family 5**

*Clinical report*

No additional clinical data available.

*Genomic analysis*

Trio exome sequencing identified a de novo missense variant in *MCM6* (NM_005915 (MCM6): c.445C>T, p.(Pro149Ser)). This variant is located in the OB-fold, has a CADD score of 28, is predicted to be deleterious by mutation taster, probably damaging by PolyPhen and deleterious by SIFT. Never reported in gnomAD.

**
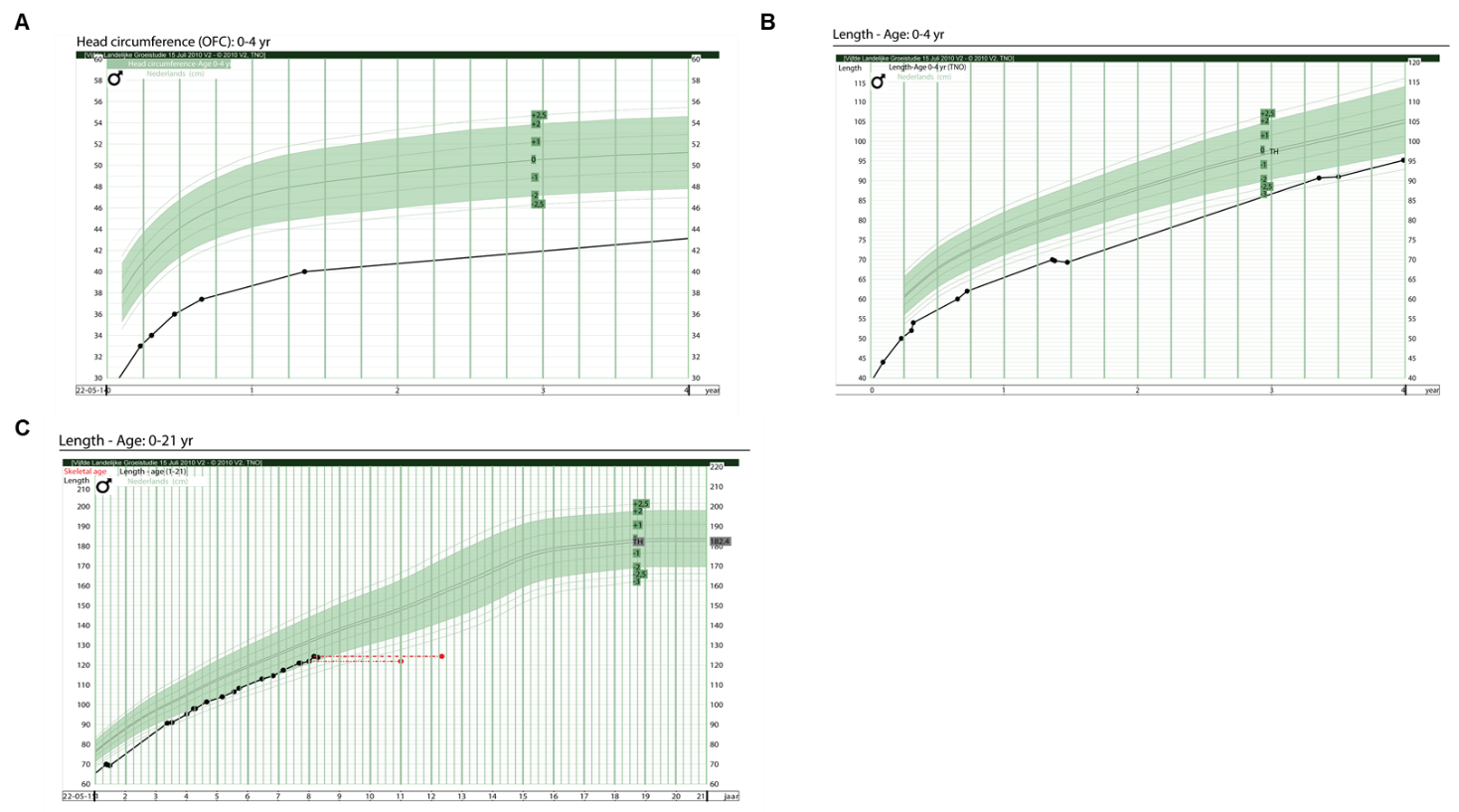
Supplemental figures**

**Fig S1 Growth charts affected individual family 1**.
Measurements of the head circumference (OFC; cm) between 0-4 years (**A**). Measurements of body length (cm) between 0-4 years (**B**) and 0-21 years of age (**C**). The red dots represent the values corrected for skeletal age.

**
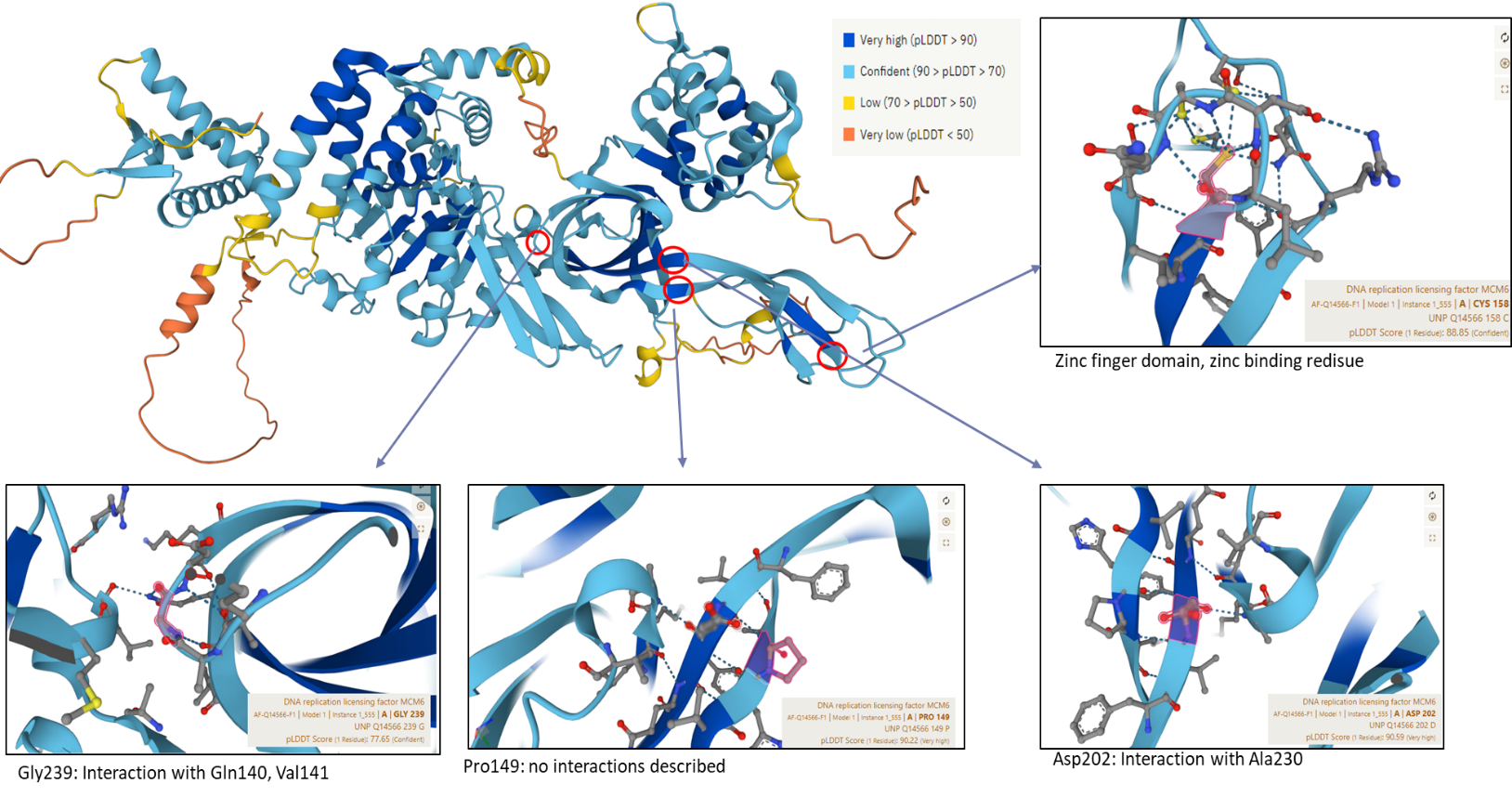
Fig S2 MCM6 protein structure and location of the missense variants.**The MCM6 protein structure was extracted from alpha fold. AlphaFold produces a per-residue confidence score (pLDDT) between 0 and 100. The residues in which missense variants were identified are shown in the magnifications, including their interactions with surrounding amino acids.

**
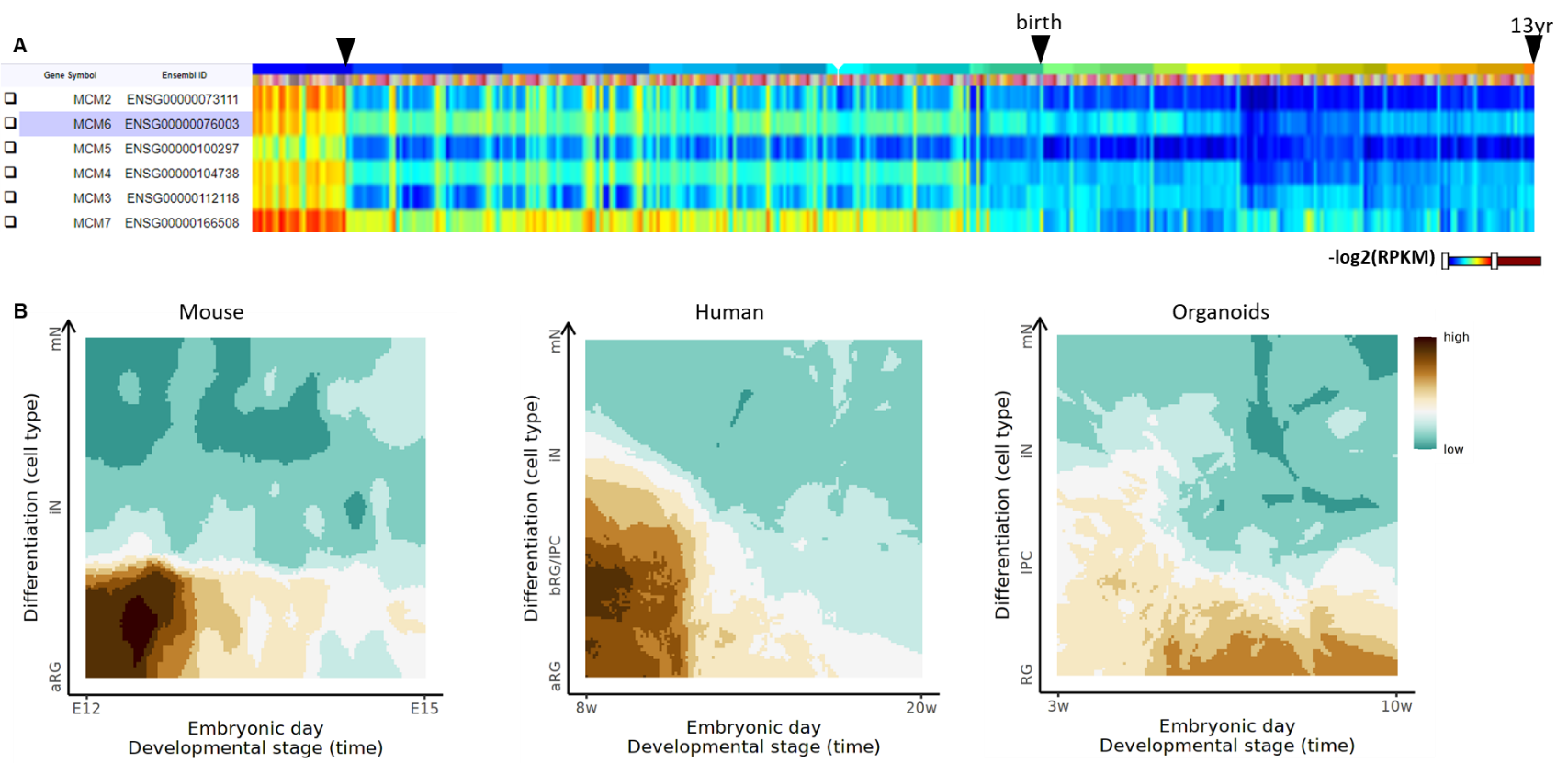
**

**Fig S3 MCM 2-7 *MCM6* expression during brain development.
(A)** Developmental transcriptome data from the Allen Brain Atlas (www.brainspan.com). Showing high expression of MCM complex genes during the first 10 post-conceptional weeks (pcw). Expression data is expressed as the reads per kilobase of transcript per million reads mapped (RPKM). (**B)** Expression of *MCM6* in mouse, human and organoid tissue during early embryonic stages (http://genebrowser.unige.ch/humous/). Showing the expression over time in different neuronal cell types (aRG: apical radial glia, bRG: basal radial glia, IPC: intermediate progenitor cells, IN: interneuron, mN: mature neuron).

**
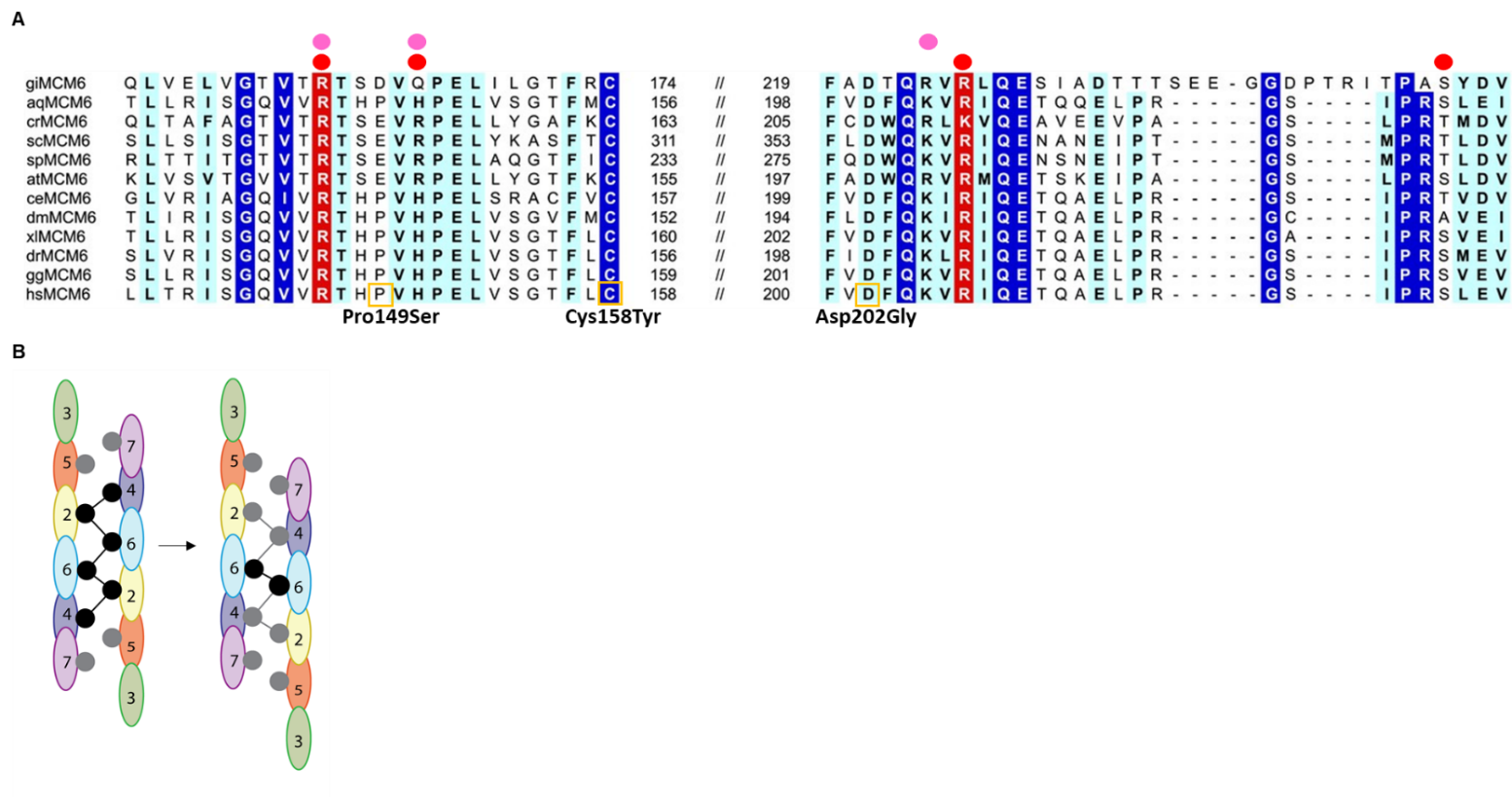
**

**Fig S4 MCM6 single stranded DNA binding domains and double hexamer formation.**Adapted from Froelich *et al^1^*. Froelich *et al* identified highly conserved regions, shared among all MCM complex components, that are thought to be involved in single stranded DNA. Amino acids depicted in dark blue are highly conserved amino acids, amino acids in light blue are highly conserved within the amino acid family. Conserved residue positions for ssDNA binding are depicted in red. Residues identified to participate directly in DNA-binding by Froelich *et al*.^1^ are shown with red dots, and those identified in work by Pucci *et al.^2^* are marked with pink dots above the sequences. The amino acids that are affected by the variants identified in individual 1,2,3&5 are marked with orange rectangles.

**Supplemental references**

1. Froelich, C.A., Kang, S., Epling, L.B., Bell, S.P., and Enemark, E.J. (2014). A conserved MCM single-stranded DNA binding element is essential for replication initiation. Elife *3*, e01993.

2. Pucci, B., De Felice, M., Rossi, M., Onesti, S., and Pisani, F.M. (2004). Amino acids of the Sulfolobus solfataricus mini-chromosome maintenance-like DNA helicase involved in DNA binding/remodeling. J Biol Chem *279*, 49222-49228.
